# Supplementary material for: The Impact of the No Jab No Play and No Jab No Pay Legislation in Australia: A Scoping Review
Source: Int J Environ Res Public Health. 2023 Jun 24;20(13):6219. doi: 10.3390/ijerph20136219 (PMC10341108; doi:10.3390/ijerph20136219)
Supplement: Supplementary file 1 [file ijerph-20-06219-s001.zip › ijerph-2292535-supplementary.pdf]

Supplementary Table S1. Characteristics of included studies.

Authors, Year, Location	Sample	Method	Legislation Evaluated	Key Findings
Armiento et al. (2020)<br><br>Melbourne, Victoria; Royal Children's Hospital (RCH)	(Parent/guardian and clinicians of children attending the Specialist Immunisation Clinic (SIC) and the Immunisation Drop in Clinic (DIC) at Royal Children's Hospital, Melbourne (RCH)<br><br>607 participants included: 395 from DIC and 214 from SIC	Three surveys:<br>SIC Parent<br>SIC Physician<br>DIC Parent<br><br>(SIC included parent and physician survey for each child)<br><br>Australian Immunisation Register (AIR) data (baseline and 7 months after clinic attendance)	No Jab No Pay<br>No Jab No Play	• Motivation to attend services due to policies at nurse-led immunisation drop-in Centre (DIC) or physician-led Specialist Immunisation Clinic (SIC):<br>⇒ No Jab No Pay = 2%<br>⇒ No Jab, No Play= 2%<br>⇒ Both NJNPay and NJNPlay = 8%<br>• Introduction of policies significantly motivated more vaccine hesitant parents (16%) compared to accepting parents (6%) with a difference of 26% (CI 16.6–34.4, $p < 0.001$).<br>• No Jab, No Play was slightly more influential in motivative parents to attend the clinics (8-9%) than No Jab No Pay (5-6%).<br>• Among parents of under-vaccinated children who planned to catch up their child's vaccine to enrol them in childcare and/or kindergarten<br>⇒ 89% vaccine accepting parents<br>⇒ 50% vaccine hesitant parents<br>⇒ 26% planning to seek medical exemption<br>⇒ 24% not planning to enrol their child.<br>• Baseline<br>Fully vaccinated = 64% (<19mths = 74%;>=19mths = 50%)<br>Partially vaccinated = 34% (<19mths = 23%;>=19mths = 49%)<br>Unvaccinated = 2% (<19mths = 3%;>=19mths = 1%)<br>• One month post clinic attendance<br>⇒ Fully vaccination = 72% (<19mths = 80%;>=19mths = 61%)<br>⇒ Partially vaccinated = 27% (<19mths = 19%;>=19mths = 39%)<br>⇒ Unvaccinated = 0.5% (<19mths = 1%;>=19mths = 0%)<br>• Seven-month post clinic attendance<br>Fully vaccinated = 68% (<19mths = 73%;>=19mths = 61%)<br>Partially vaccinated = 32% (<19mths = 27%;>=19mths = 39%)<br>Unvaccinated = 0% (<19mths = 0%;>=19mths = 0%)<br><br>• Compared to baseline, full vaccination rates were higher for 1 month post attendance for parent motivated by the policies (40%) or vaccine hesitant (35%).

Attwell et al. (2020)<br><br>Australia (National data)	Australian Immunisation Register (AIR) data for children ages 12, 24 and 60 months<br><br>Deidentified AIR data 31 March, 2019 covering the period 2009-2017 for children ages 12, 24 and 60 months	Time-series analysis	No Jab No Pay<br>No Jab No Play	• Due to changes to coverage assessment for full coverage at 24 months it was not possible to test for policy-related changes for this age group.<br>• Rates of children not fully vaccinated at 12 months and 5 years steadily declined from 2009 to 2017<br>• In all states/territories there was a larger annual rate of decrease of 12 months and 5-year old's not fully vaccinated after policy intervention, however this was not significant<br>• However, for New South Wales, vaccine coverage rates were static prior to policy intervention, but reflected an annual increase in coverage rates of 1.25% after policy implementation (p = 0.001).<br>• The impact of the policies was indistinguishable between communities with high, medium and low numbers of registered vaccine refusers.<br>• Difficult to discern specific policy impact with the implementation of the Federal NJNPay policy in 2016 and staggered implementation of NJNPlay (e.g. NSW, 2014; Victoria 2016)
Centre for Evaluation and Research Evidence (2020)<br><br>Victoria	AIR data (2013-20)<br>Department of Education and Training data (2013–20)<br>Kindergarten Information Management System and Victorian Child and Adolescent Monitoring System quantitative data (n = 1,062 surveys)<br>– parent/carer survey (n = 440)<br>– immunisation provider survey (n = 266)<br>– early childhood education and care service provider survey (n = 356)<br>Qualitative data	Mixed methods:<br>Document analysis<br>Literature review<br>Analysis of data-bases<br>Surveys<br>Interviews	No Jab No Play	• Increase in immunisation rate since introduction of the No Jab No Play legislation in Victoria (2016)<br>⇒ 1 year old = 3.4%<br>⇒ 2 year old = 3.7%<br>⇒ 5 year old = 3.3%<br>• Healthcare providers (84%) acknowledged that legislation led to increased vaccine uptake.<br>• Vaccine hesitant parents:<br>⇒ Influenced by the legislation and peak increase in vaccination rate observed when legislation was introduced.<br>⇒ Immunisation providers (74%) reported that reputable and accurate information to be provided to parents/carers with vaccine safety concerns.<br><br>• Aboriginal and Torres Strait Islander Immunisation coverage high and exceeded the target for 1-year-old (96.8 per cent) and 5-year-olds (97.9 per cent) Immunisation rates for Aboriginal and Torres Strait Islander children aged 2 years is steadily increasing and is 92.6 per cent, but still 2.4 per cent below the target rate

	– key informant interviews (n = 43)<br>– parents and carers, ECECS providers, immunisation providers, immunisation and education experts, peak bodies, migrant services, and Aboriginal community-controlled organisations<br>– written submissions (n = 9) key stakeholder organisations and peak bodies.			
Cordingley et al. (2021)<br><br>New South Wales (one rural medical practice data)	All patients aged 21 months to ≤ 19 years at the practice who received a national immunisation program (NIP) vaccination between 2012-17 (cohort: n = 6,422; n = 2,133 NIP vaccine records; n = 453 catch up vaccinations)	Retrospective clinical audit: single medical practice<br><br>Data collected 9th January 2018<br><br>Vaccinations considered overdue if given 3 months after the due date	No Jab No Pay	• Focused on the impact of NJNPay on catch-up vaccinations.<br>• Incidence of annual catch-up vaccinations for the year 2012 – 2015 was on average 6.2% pre-policy implementation.<br>• An increase from 6.2% to 9.2% in 2016 ($p < .001$) and 7.8% in 2017 ($p = .027$) was observed post-policy implementation.<br>• Age as a secondary outcome measurement of catch-up vaccination incidence rates before policy implementation showed statistically significant:<br>⇒ increases for children:<br>8–11 years (3.2%–5.6%, $p = .038$)<br>12–15 years (7.5%–14.7%, $p < .001$)<br>16–19 years (3.3%–10.2%, $p < .001$)<br>⇒ reduction in children aged 1–3 years (11.4%–6.2%, $p = .015$)<br>• Vaccination type as a secondary outcome measurement of catch-up vaccination incidence rates before policy implementation showed statistically significant catch-up rates for DTPa (1.6%-2.4%, $p = 0.022$)
Frawley et al. (2018)<br><br>Australia	Australian parents with at least one child <6 years<br>N= 429	Cross- sectional survey: Online, self complete	No Jab No Pay	• Influence of information sources on vaccination.<br>⇒ GPs (78.3%), friends/family (55.7%), nurses/midwives (43.4%), government websites (33.3%), paediatricians (30.5%), parenting groups (16.8%), non-government websites (13.1%), newspapers/magazines (8.4%), CM-practitioners (6.3%) and pharmacists (4.4%)<br>⇒ Significant relationship between vaccine uptake and being influenced by a paediatrician ($p = .001$) or GP ($p = .019$).

				⇒ significant relationship between child vaccination status not being up-to-date and being influenced by a CM-practitioner (p < .001), non-government website (p = .002) or information source other than those listed (p< .001).<br><br>• Introduction of NJNPay and influences on parents' decision to vaccinate<br>⇒ 2.6% of parents had their child immunised<br>⇒ 3.9% stated had no effect<br>⇒ 1.2% said it had made them less likely to vaccinate<br>⇒ 1.2% of parents stated they are considering vaccination due to the legislation.
Helps, Leask & Barclay (2018)<br><br>New South Wales (Bryon Region)	Non-vaccinating parents (n = 31)	Qualitative: semi-structured face-to-face interviews	No Jab No Pay	• Impact of legislative changes on families was minimum due to:<br>⇒ Ineligibility based on income<br>⇒ their choice not to receive government payments for which they were eligible and/or<br>⇒ low utilisation of childcare services<br><br>• Strategies suggested by families who are currently, or expecting to be, affected by the inability to lodge exemptions and receive family tax benefits and childcare subsidies include:<br>⇒ increasing extended family support<br>⇒ reducing work and study commitments<br>⇒ informal childcare arrangements, house sharing or relocating to reduce rental burden<br>⇒ withdrawal of children from childcare, including before and after school care services.<br><br>• Participants reported that the government measure would not change their mind about vaccinating their children.<br><br>• Concerns were expressed about further punitive for those declining vaccination and home schooling or accessing education outside the mainstream system were reported as solutions.
Hull et al. (2020)<br><br>Australia (National data)	Participants: AIR data for children 5 to < 7 years; 7 to < 10 years; 10 years to < 20 years	**Cross sectional** analysis of AIR data on catch-up vaccination<br>AIR data from 1 January 2013-31 December 2014 (baseline, prior to NJNPay)	No Jab No Pay	• NJNPay and catch-up vaccination explored<br>• Ages 5 to less than 7 years:<br>⇒ Proportion of DTPa3 catch-up vaccination after introduction of No Jab No Pay was 15.5% (3,366 of 35,673 unvaccinated children) indicating an increase from 9.4% at baseline.

		and 1 December 2015- 31 December 2017<br><br>Analyse catch-up vaccination rate for third dose of DTPa3 and first dose of MMR1at baseline. Catch-up for selected vaccination at 5 to less than 7 years; 7 to les s than 10 years and 10 to less than 20 years. MMR2 catch-up vaccinations by socio-economic status; Indigenous status and geographic characteristics. Analyses conducted by age group, geographical location (remote or metropolitan), socio-economic status and Aboriginal and Torres Strait Islander status.		⇒ Lower catch-up rates in remote (18.3%) and regional (21.5%) compared to metro areas (22.7%).<br>• Ages 7 to less than 10 years:<br>⇒ 22.3% received MMR2 and 12.5% received DTPa3 vaccination catch up during first two years of No Jab No Pay.<br>⇒ Greater increase for those with lowest socio-economic status (32.3%) compared to highest status areas (14.6%).<br>⇒ Greater increase indigenous (43.4%) compared to non-indigenous Australians (21.9%).<br>• Ages 10 to less than 20 years:<br>⇒ 17.6% received MMR2 and 8.8% received dTpa3 vaccination catch up during first two years of No Jab No Pay.<br>⇒ Greater increase for those with lowest socio-economic status (29.1%) compared to highest status areas (7.6%).<br>⇒ Greater increase indigenous (35.8%) compared to non-indigenous Australians (17.1%).<br>⇒ Lower catch-up rates in remote (15.8%) and metro areas (17.1%) compared to regional (19.5%) areas.
Li and Toll (2021)<br><br>Australia (National data)	AIR data for children aged <= 6 years	Generalised linear models on data 2014-2018 Statistical Area 3	No Jab No Pay<br>No Jab No Play	• Removal of philosophical or religious exemptions was associated with increases in vaccine coverage:<br>⇒ Approx. 2-4% increase for one years;<br>⇒ Approx. 1-1.5% increase for two years;<br>⇒ Approx. 1-3.5% increase for five years<br><br>Greater improvement in coverage was found in areas with greater socioeconomic disadvantage, lower median income, more benefit dependency, and higher pre-policy baseline coverage
Toll and Li (2022)<br><br>Australia (National data)	AIR data (1-, 2-, 5-year-olds), linked to regional characteristics from the Australian Bureau of Statistics at the statistical area 3 (SA3) level	Data compared between January 2016 and December 2019<br><br>Intervention and control and before and after study design	No Jab No Play	• Increases in coverage significant, however small at around 1% across age groups<br>• Increases greater for 2-and 5-year-olds<br>• Considering aggregate time trends and regional characteristics improvement in full coverage poste year 1 and 2 of policy implementation:<br>• Age one (post-year 1: 0.15%; post-year 2: 0.56%)<br>• Age two (post-year 1: 0.49%; post-year 2: 1.15%)

		NSW and WA data were used for the main estimation using a quasi-experimental design. NJNPLay was introduced (without consinetious objections) in NSW and WA in 2018 and 2019 respectively.<br>Analysis for Queensland and Victoria where NJNPay and NJNPlay were both implemented in 2016 employed a repeated measures group modelling approach. Other States/Territories acted as control		• Age five (post-year 1: 0.38%; post-year 2: 0.71%)<br>• Policy effect was insignificant and dispersed the lowest quantiles of disadvantage<br>• Policy effect was smaller and insignificant in the highest socio-economic areas
Trent et al. (2019)<br><br>Australia	Parents with children < 5 years (n = 411)	Online survey distributed by Survey Sampling International (SSI)<br>Randomly distributed to SSI panel members to achieve a representative sample in terms of gender and state/territory residence	No Jab No Pay	• Support for No Jab No Pay<br>⇒ Supportive of the policy (82%)<br>⇒ Females were twice as likely as males to support the policy (OR = 2.14, 95% CI [1.28, 3.61], $p < 0.01$), but no difference observed after controlling for disease risk perception and needing the financial incentives (AOR 1.62, 95% CI [0.92, 2.84], $p = 0.09$)<br>⇒ Demographics status such as age, country of birth, education level, Aboriginal/Torres Strait Islander status, or state/territory of residence did not affect the results<br>⇒ No significant difference in supporting the policy observed between those who utilise childcare services compared to those who don't<br>⇒ For parents that reported needing the financial incentive to afford their family expenses, the odds of supporting the policy were more than twice than those who don't (OR = 2.14, 95% CI = [1.24, 3.68], $p < 0.001$)<br>• Increased willingness to vaccinate as a result of the legislation 32%<br>• Willingness to vaccinate<br>⇒ To reconsider vaccination were more than nine times higher among respondents that reported needing the financial incentives (AOR = 9.66, 95% CI = [4.98, 18.72], $p < 0.001$).<br>⇒ Parents that reported utilising child care centres or family day care had more than twice the odds of being willing to reconsider

				vaccination compared to parents that did not (AOR = 2.09, 95% CI = [1.04, 4.17], p = 0.04).<br>⇒ Victoria respondents had higher odds of reconsidering vaccination compared to those from New South Wales and other states (AOR = 2.32, 95% CI = [1.00, 5.38], p = 0.05)<br>⇒ Demographics status such as age, country of birth, education level, Aboriginal/Torres Strait Islander status, or state of residence did not affect the results
